# Utility of Kansas City Cardiomyopathy Questionnaire (KCCQ) in Assessing Quality of Life among Patients with Heart Failure Undergoing Exercise Training Rehabilitation: A Systematic Review

**DOI:** 10.3390/diseases12040064

**Published:** 2024-03-22

**Authors:** Ilona Emoke Sukosd, Silvius Alexandru Pescariu, Cosmin Faur, Alexandra Ioana Danila, Catalin Prodan-Barbulescu, Ovidiu Fira-Mladinescu

**Affiliations:** 1 Doctoral School, Department of General Medicine, “Victor Babes” University of Medicine and Pharmacy Timisoara, 300041 Timisoara, Romania; sukosd.emoke@umft.ro (I.E.S.); catalin.prodan-barbulescu@umft.ro (C.P.-B.); 2 Center for Research and Innovation in Precision Medicine of Respiratory Diseases, “Victor Babes” University of Medicine and Pharmacy Timisoara, Eftimie Murgu Square 2, 300041 Timisoara, Romania; mladinescu@umft.ro; 3 Department of Cardiology, “Victor Babes” University of Medicine and Pharmacy Timisoara, 300041 Timisoara, Romania; pescariu.alexandru@umft.ro; 4 Department of Orthopedics, “Victor Babes” University of Medicine and Pharmacy Timisoara, 300041 Timisoara, Romania; 5 Department of Anatomy and Embriology, Discipline of Pulmonology, “Victor Babes” University of Medicine and Pharmacy Timisoara, Eftimie Murgu Square 2, 300041 Timisoara, Romania; alexandra.danila@umft.ro; 6 IInd Surgery Clinic, “Victor Babes” University of Medicine and Pharmacy Timisoara, Eftimie Murgu Square 2, 300041 Timisoara, Romania; 7 Department of Infectious Diseases, Discipline of Pulmonology, “Victor Babes” University of Medicine and Pharmacy Timisoara, Eftimie Murgu Square 2, 300041 Timisoara, Romania

**Keywords:** quality of life, heart failure, cardiac rehabilitation, exercise training

## Abstract

This systematic review evaluates the effectiveness of the Kansas City Cardiomyopathy Questionnaire (KCCQ) in assessing quality of life improvements among patients with heart failure (HF) undergoing various forms of exercise training rehabilitation, including telemedicine and in-person modalities, across all stages of HF, irrespective of ejection fraction (EF) and clinical status. The aim was to collate evidence from studies employing the KCCQ as a measure of quality of life (QoL). A comprehensive search strategy was implemented across PubMed, Scopus, and Embase databases, adhering to the PRISMA guidelines, including literature up until October 2023. Inclusion criteria encompassed studies on patients diagnosed with HF undergoing exercise training rehabilitation assessed by KCCQ. Nine articles met the inclusion criteria, involving a total of 3905 patients from various global locations and conducted between 2012 and 2022. Results indicated significant heterogeneity in exercise interventions and patient characteristics. Notably, high-intensity interval training (HIIT) showed a marked improvement in KCCQ scores (from 68.0 to 80.0) compared to moderate continuous training (MCT) and control groups, underscoring its potential for enhancing QoL. Additionally, a significant improvement in the 6-min walking test (6MWT) outcomes was observed, with an average increase of 106 m (95% CI: 60, 152) in one study, reflecting physical capacity enhancements. However, the difference in KCCQ scores between intervention and control groups was not statistically significant in several studies. In conclusion, the KCCQ’s effectiveness is highlighted by its ability to detect clinically meaningful improvements in QoL across diverse exercise modalities, including HIIT and MCT, tailored to the specific needs of HF populations. The consistent correlation between KCCQ score improvements and enhanced physical outcomes, such as the 6MWT, supports its reliability in capturing the nuanced benefits of exercise interventions on patient well-being.

## 1. Introduction

The management of heart failure (HF) and its complications has evolved significantly over the past decades, with an increasing emphasis on improving patient-centered outcomes, particularly quality of life (QoL) [1,2]. Heart failure, a chronic, progressive condition that is commonly associated with multiple respiratory comorbidities, not only impairs physical function but also profoundly impacts emotional and social wellbeing [3,4,5,6]. As such, the assessment of QoL of life becomes a pivotal component of comprehensive management of HF. Among the tools developed for this purpose, the Kansas City Cardiomyopathy Questionnaire (KCCQ) has been widely recognized for its sensitivity and specificity in measuring the health status of HF patients [7]. However, the effectiveness of the KCCQ in capturing the QoL improvements attributable to exercise training rehabilitation remains inadequately explored.

Exercise training rehabilitation has emerged as a cornerstone in the management of HF, with evidence supporting its role in enhancing physical capacity, symptoms, and overall QoL [8,9,10]. In recent years, and notably during the COVID-19 pandemic, there has been a surge in the adoption of varied rehabilitation modalities, including telemedicine-based interventions, to address the limitations imposed by traditional in-person therapy sessions [11,12,13]. This period has underscored the flexibility and potential of exercise rehabilitation programs to be tailored to individual patient needs, as well as in the context of associated infections, thereby maximizing therapeutic benefits [14,15].

Despite the recognized value of exercise training, the measurement of its impact on QoL presents a challenge, attributed to the diversity of assessment tools, where each of these instruments evaluates different domains of QoL, making direct comparisons problematic [16,17]. This heterogeneity in measurement complicates the aggregation of data across studies, thereby hindering a cohesive understanding of the effects of exercise rehabilitation on the QoL among HF patients. The KCCQ, with its comprehensive assessment of physical limitation, symptoms, self-efficacy, social interference, and q QoL, offers a potential solution to this challenge.

Given the variety of exercise rehabilitation modalities and the nuanced impacts these interventions can have on different aspects of a patient’s life, a thorough examination of the KCCQ’s performance across these dimensions is warranted. Therefore, the hypothesis of this systematic review was that the KCCQ effectively measures the QoL improvements in patients with HF participating in exercise training rehabilitation programs, including those delivered via telemedicine, and in all stages of HF, regardless of ejection fraction (EF) and clinical status. The aims and objectives of this study were to collate all studies and evaluate the application of the KCCQ in this context to compare its efficacy with other QoL assessment tools. By achieving these objectives, this review intends to contribute to the optimization of HF management practices, ensuring that they are both effective and patient centered.

## 2. Materials and Methods

### 2.1. Protocol and Registration

To ensure a comprehensive and systematic review of the literature, this study employed a detailed search strategy across multiple key electronic databases, including PubMed, Scopus, and Embase. The search aimed to encompass literature published up until October 2023 to include the most current research available on the topic.

The search strategy was carefully developed to include a wide array of keywords and phrases pertinent to the study’s goals, focusing on the evaluation of QoL in patients with HF undergoing exercise training rehabilitation. Key search terms included: “heart failure”, “cardiac rehabilitation”, “exercise training”, “KCCQ”, “Kansas City Cardiomyopathy Questionnaire”, “quality of life”, “QoL”, “telemedicine”, “remote rehabilitation”, “physical activity”, “patient-reported outcomes”, “health-related quality of life”, “HRQoL in cardiovascular diseases”, “effectiveness of exercise rehabilitation”, and “telehealth interventions for heart failure”.

Boolean operators were utilized to refine and combine the search terms effectively. The search string was designed as follows: (“heart failure” OR “cardiac dysfunction” OR “ventricular dysfunction” OR “HF”) AND (“cardiac rehabilitation” OR “exercise training” OR “physical rehabilitation” OR “exercise therapy” OR “rehabilitation program” OR “exercise intervention”) AND (“Kansas City Cardiomyopathy Questionnaire” OR “KCCQ” OR “quality of life assessment” OR “QoL measurement” OR “HRQoL” OR “patient-reported outcome measures” OR “PROMs”) AND (“telemedicine” OR “telehealth” OR “remote rehabilitation” OR “digital health” OR “online therapy” OR “virtual rehabilitation”) AND (“physical activity” OR “aerobic exercise” OR “resistance training” OR “endurance training” OR “physical fitness”) AND (“psychosocial factors” OR “mental health” OR “emotional well-being” OR “social support”) AND (“home-based rehabilitation” OR “clinic-based rehabilitation” OR “hybrid rehabilitation” OR “community-based exercise”) AND (“engagement strategies” OR “patient adherence” OR “motivational interviewing” OR “self-management”).

In adherence to the preferred reporting items for systematic reviews and meta-analyses (PRISMA) guidelines, this systematic review protocol ensures a structured, transparent, and reproducible approach to methodology [18]. Additionally, to promote transparency and accessibility of the research process and findings, the review has been registered with the Open Science framework (OSF) to ensure open access to our methodology and findings, with the registration code osf.io/d73vm.

### 2.2. Eligibility Criteria

The inclusion criteria for this systematic review were established as follows: (1) studies must involve patients diagnosed with HF, regardless of the etiology or stage of the disease; (2) research must focus on exercise training rehabilitation programs for patients with HF, including any form of structured physical activity, such as aerobic exercise, resistance training, or combined exercise modalities, delivered through various formats including in-person, remote (telemedicine), or hybrid approaches; (3) studies should assess the QoL using the KCCQ; (4) inclusion of randomized controlled trials, observational studies, clinical trials, cohort studies, case-control studies, and cross-sectional studies that provide clear and detailed methodologies regarding the assessment of QoL in patients with HF undergoing exercise training rehabilitation; (5) only peer-reviewed articles published in English are to be included.

The exclusion criteria were defined to refine the scope of the review and ensure the relevance and quality of the included studies: (1) research not involving human participants, such as in vitro or animal model studies, were excluded; (2) studies not examining the QoL specifically in patients with HF undergoing exercise training rehabilitation, or not utilizing the KCCQ or similar validated instruments for QoL assessment; (3) studies that do not provide clear, quantifiable outcomes related to QoL measures post-intervention, or lack sufficient detail for a comprehensive analysis; (4) exclusion of grey literature, including non-peer-reviewed articles, preprints, conference proceedings, general reviews, commentaries, and editorials, to maintain the credibility and reliability of the data included in the review.

### 2.3. Definitions

The latest guidelines from the American Heart Association (AHA), the American College of Cardiology (ACC), and the European Society of Cardiology (ESC) provide detailed classifications and definitions of HF, recognizing its diverse etiologies, pathophysiology, and clinical presentations [19].

The New York Heart Association (NYHA) classification is a widely used system to describe the functional limitations of patients with HF [20]. It categorizes patients into four classes based on their symptoms and physical activity limitations:

NYHA Class I: No limitation of physical activity. Ordinary physical activity does not cause undue fatigue, palpitation, or dyspnea.
NYHA Class II: Slight limitation of physical activity. Comfortable at rest, but ordinary physical activity results in fatigue, palpitation, or dyspnea.
NYHA Class III: Marked limitation of physical activity. Comfortable at rest, but less than ordinary activity causes fatigue, palpitation, or dyspnea.
NYHA Class IV: Unable to carry on any physical activity without discomfort. Symptoms of HF at rest; if any physical activity is undertaken, discomfort increases.

Studies included in the current review can include both compensated and uncompensated HF, as well as preserved or reduced ejection fraction HF: (1) heart failure with reduced ejection fraction (HFrEF), previously known as systolic HF, is defined by a left ventricular ejection fraction (LVEF) of less than 40%; (2) heart failure with preserved ejection fraction (HFpEF), also known as diastolic heart failure, is diagnosed when symptoms of HF are present but the left ventricular ejection fraction is greater than or equal to 50%; (3) heart failure with mid-range ejection fraction (HFmrEF): This category includes patients with HF symptoms and a left ventricular ejection fraction that is neither clearly preserved nor reduced, typically ranging from 41% to 49%. This category acknowledges patients who fall into a gray area and may share characteristics of both HFrEF and HFpEF.

### 2.4. Data Collection Process

The process for selecting studies for this systematic review began with the removal of duplicates, followed by a thorough assessment of abstracts by two independent researchers. This initial screening aimed to determine each study’s relevance to the review’s objectives and adherence to predefined inclusion criteria. Any disagreements in study selection were resolved through discussion with a third researcher, ensuring a collaborative and consensus-based approach.

In the context of this systematic review focusing on the assessment of the KCCQ survey among patients with HF undergoing exercise training rehabilitation, the initial search across databases resulted in a total of 1858 articles. After a screening process, 583 articles were selected for closer examination, while 315 were identified as duplicates and subsequently excluded to streamline the review process. The screening phase involved a detailed evaluation of abstracts to determine relevance to the study’s objectives, conducted independently by two reviewers (A.I.D. and C.P-B.), with any discrepancies resolved through consultation with a third reviewer (S.A.P.) to maintain the integrity and objectivity of the selection process.

**Figure 1:**
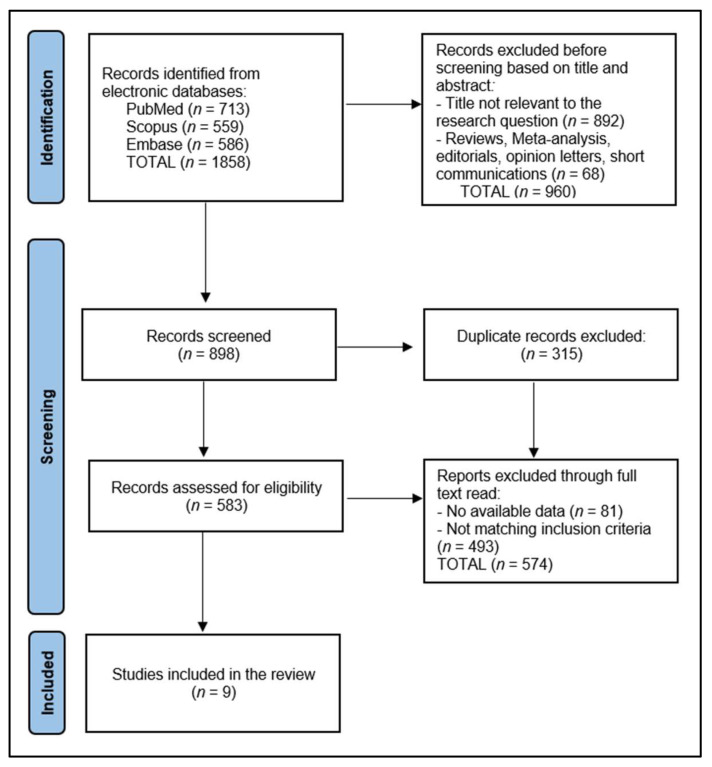
PRISMA flow diagram.

Ultimately, 9 articles met the inclusion criteria established for this review, as presented in Figure 1. This selection was based on a comprehensive evaluation of each article’s content, focusing on the utilization of the KCCQ survey to gauge QoL and physical improvements after exercise training rehabilitation in patients with HF. The included studies were subject to an in-depth data extraction phase, handled by two dedicated researchers, in order to gather and synthesize information pertinent to the study designs, participant demographics, specifics of the rehabilitation programs, application of the KCCQ survey, and the resultant QoL outcomes.

### 2.5. Quality Assessment

To assess the quality of the studies included in our review, the Newcastle–Ottawa Scale was utilized for evaluating cohort studies and the Cochrane Collaboration’s tool for assessing randomized trials [20]. Two researchers independently conducted the evaluations, assigning scores that reflect the studies’ quality as either low, medium, or high. This method facilitated a neutral assessment of the literature under review, providing a solid foundation for the systematic analysis.

## 3. Results

### 3.1. Study Characteristics

The systematic review analyzed a total of nine studies [21,22,23,24,25,26,27,28,29], detailed in Table 1, spanning various countries including the United States [21,22,23,25,27], Germany [24,26], Taiwan [28], and Japan [29], conducted between 2012 and 2022. This diverse collection of research emphasizes a widespread interest in the application of the KCCQ to assess the QoL in patients with HF undergoing exercise training rehabilitation. The studies predominantly utilized randomized trial designs, with six out of nine studies [22,23,24,25,27,29] employing this methodology, indicating a strong preference for rigorous experimental control to evaluate the efficacy of exercise interventions. Notably, the research settings varied, encompassing both individual and group-based rehabilitation programs, reflecting the multifaceted approach to exercise training in HF care.

The quality of evidence presented across these studies was generally high, with five studies [22,23,24,25,27] receiving high-quality ratings. This suggests that the majority of included research provided robust and reliable results, underpinned by well-designed trials that likely included randomization, control groups, and adequate blinding. Meanwhile, the prospective and retrospective cohort studies [21,26,28], along with one randomized trial [29], were assessed as medium quality, indicating a moderate level of evidence reliability. 

### 3.2. Participants’ Characteristics

The results from Table 2 provide a comprehensive overview of participant characteristics from nine studies that collectively encompassed 3905 patients, highlighting a substantial body of research into this intervention’s impact. Age and sex distribution varied across studies, reflecting a broad spectrum of HF populations. For instance, the average age ranged from 56 years in the intervention group of Norman et al. [21] to 73.1 years in the intervention group of Kitzman et al. [25]. The proportion of men also varied significantly, from as low as 20% in the intervention group of Kitzman et al. [22] to as high as 84.3% in the intervention group of Chen et al. [28], underscoring the gender diversity in these studies.

Interventions across these studies were aimed at patients with different HF characteristics, including those with preserved ejection fraction (HFPEF), as seen in the study by Kitzman et al. [22], and those undergoing high-intensity interval training (HIIT) or moderate continuous training (MCT), as observed in Mueller et al. [24]. This diversity in intervention types points to the varied approaches in exercise training rehabilitation for patients with HF.

Left ventricle ejection fraction (LVEF) in the intervention group of Norman et al. [21] was 34%, whereas it was significantly higher at 60% in both the intervention and control groups of Kitzman et al. [22], indicating a focus on patients with differing severities of HF, some studies including only HFpEF patients [22,25,27]. Also, BMI varied across the intervention and control groups, reflecting the diverse physical profiles of the participants. In the study by Norman et al. [21], both the intervention and control groups had similar BMIs, with values of 33.0 and 33.2 kg/m^2^, respectively, indicating a consistent selection criterion for obesity or overweight status among participants. Contrastingly, Kitzman et al. [22] reported higher BMI values in the intervention group (40.3 kg/m^2^) compared to the control group (38.4 kg/m^2^), emphasizing the focus on obese patients with HF with preserved ejection fraction (HFPEF), a subgroup known to benefit from specific exercise regimens.

Baseline BNP and NT-proBNP levels were essential in understanding the participants’ initial cardiac function. The study by Parikh et al. [23] reported significantly high NT-proBNP levels in both the intervention (716 pg/mL) and control groups (839 pg/mL), illustrating the severity of HF among participants. Similarly, Kitzman et al. [25] provided a detailed analysis of both BNP and NT-proBNP levels, with the intervention group showing baseline BNP levels of 595 pg/mL and NT-proBNP levels of 2527 pg/mL, further highlighting the clinical severity of the study population.

Control groups across these studies were not uniform in their composition or the nature of the control condition. For instance, the control group in Norman et al. [21] received attention control, a common methodology to ensure engagement without the specific benefits of exercise intervention. In contrast, the study by Mueller et al. [24] compared HIIT and MCT against a no-training control, providing a nuanced understanding of how different exercise intensities impact HF outcomes. The variety in control conditions across studies points to the methodological diversity in researching the effects of exercise training on HF, crucial for isolating the specific benefits of exercise interventions.

### 3.3. Rehabilitation Program Characteristics

Table 3 outlines the characteristics of rehabilitation programs across nine studies focused on exercise training rehabilitation for patients with HF. These programs varied significantly in duration, frequency, and intensity of training, reflecting the tailored approaches to meet the specific needs of HF populations. For example, the training time followed by the studies ranged from 3 months, as seen in Kitzman et al. [25], Murray et al. [27], Chen et al. [28], and Nagatomi et al. [29], to 2.5 years in Parikh et al. [23], the longest duration noted.

Frequency of training also showed variability, with most studies opting for aerobic exercises three days per week [21,22,24,25,27,28], a schedule supported by clinical guidelines for patients with HF. However, Mueller et al. [24] differentiated between HIIT three days per week and MCT five days per week, illustrating the study’s exploration of exercise intensity’s impact on HF rehabilitation.

The rehabilitation program descriptions provided detailed insights into the types of exercises prescribed and their intended intensity levels. Aerobic exercises were commonly used, with intensities often set to a percentage of heart rate reserve (HRR) or based on the Rating of Perceived Exertion (RPE) scale. For instance, Norman et al. [21] included aerobic exercises at 40% to 70% HRR and resistance training targeting both upper and lower extremities, designed to enhance cardiovascular fitness and muscle strength. Similarly, Kitzman et al. [22] focused on walking, leveraging individualized prescriptions to optimize outcomes based on test results, illustrating a personalized approach to exercise therapy.

Innovatively, Parikh et al. [23] initiated a combined supervised and home-based program, starting with 36 supervised sessions followed by two years of home training, acknowledging the importance of long-term, sustainable exercise habits. Meanwhile, HIIT and MCT programs in Mueller et al. [24] were specified with clear intensity targets, providing evidence of the structured and rigorous nature of these interventions.

### 3.4. Survey Results

Table 4’s examination of the Kansas City Cardiomyopathy Questionnaire (KCCQ) and 6 Minute Walk Test (6MWT) outcomes provides a quantitative view through the impact of exercise training on patients with HF. For the KCCQ scores, significant variations were noted among the studies. In the case of Norman et al. [21], the intervention group’s KCCQ score improved from 69.7 at baseline to 81.0 at finish, while the control group’s score went from 72.8 to 77.9. Despite these improvements, the difference between the groups was not statistically significant, indicating that exercise did not lead to a unique improvement in QoL over the control condition. On the other hand, Mueller et al. [24] showed a significant difference in KCCQ scores post-intervention, with HIIT participants increasing from 68.0 to 80.0 compared to MCT participants who went from 62.2 to 77.0 and controls from 65.7 to 72.0, highlighting the superior effectiveness of HIIT in enhancing QoL.

The 6MWT outcomes also provide insight into the physical benefits of the exercise interventions. For instance, Kitzman et al. [22] reported a statistically significant difference in 6MWT outcomes, with an improvement of 106 m (95% CI: 60, 152) in the intervention group, underscoring the substantial impact of exercise on physical capacity. Similarly, Murray et al. [27] documented significant improvements in 6MWT distances, with the intervention group for diabetes mellitus (DM) patients improving from 183 m at baseline to 281 m at finish and the non-DM intervention group from 209 to 286 m, demonstrating the wide-reaching benefits of exercise across different subsets of patients with HF.

Interestingly, Kitzman et al. [25] observed a notable increase in KCCQ scores from 40 to 69 in the intervention group, and from 42 to 62 in the control group, alongside significant improvements in the 6MWT, with the intervention group maintaining nearly the same distance (194 m at baseline to 193 m at finish) despite an expected decrease due to HF progression. This contrasts with the control group, which saw a decrease from 293 m at baseline to 260 m at finish, suggesting that exercise not only halted but marginally reversed the decline in physical performance.

**Figure 2:**
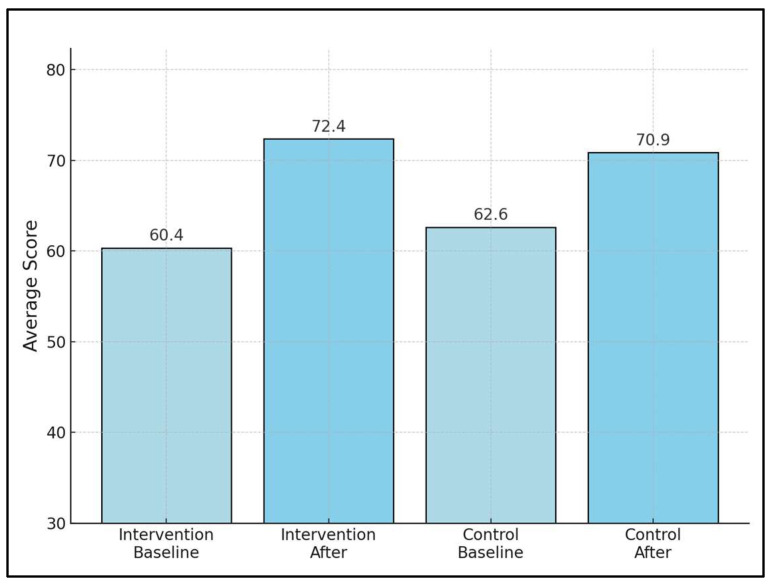
Average KCCQ scores comparison between intervention and control groups.

Chen et al. [28] reported a significant improvement in KCCQ scores, with the intervention group experiencing a 32.9-point increase compared to a 20.3-point increase in the control group, indicating a marked benefit of exercise on QoL, even though specific baseline and finish scores were not reported. The average KCCQ scores at baseline in intervention and control groups were 60.4 and 62.4, respectively, compared to 72.4 and 70.9 after intervention, as seen in Figure 2.

## 4. Discussion

### 4.1. Summary of Evidence

The systematic review found important insights into the effectiveness of exercise-based cardiac rehabilitation (ExCR) programs across diverse HF populations. Central to this review is the evaluation of KCCQ scores, which serve as a primary measure of QoL improvements following exercise interventions. The aggregated data from various studies, involving a total of 3905 participants, provide a substantial basis for understanding how exercise impacts the perceived QoL of patients with HF.

Key findings reveal that exercise training, varying in intensity from HIIT to MCT, and spanning durations from 3 months to 2.5 years, demonstrates a potential to improve QoL among patients with HF. Notably, programs that incorporated HIIT showed a significant improvement in KCCQ scores, suggesting that higher intensity workouts might be more effective in enhancing the QoL. However, the variability in outcomes across different studies indicates that the response to exercise training is highly individualized, with factors such as the baseline severity of HF, patient age, sex, and baseline functional capacity playing crucial roles in determining the effectiveness of exercise interventions [30].

The review underscores the complexity of exercise rehabilitation in HF care, highlighting the need for personalized exercise prescriptions tailored to the specific clinical and functional profiles of patients. Despite the variability in outcomes, the consistent use of KCCQ as a measure across studies reinforces its utility in assessing QoL improvements, providing a valuable tool for clinicians to gauge the impact of exercise rehabilitation programs. This systematic review, therefore, not only sheds light on the positive effects of exercise training on the QoL among patients with HF but also calls for further research to refine exercise prescriptions to maximize patient benefits.

The systematic review, alongside the studies by Taylor et al. [31] and Piotrowicz et al. [32], collectively emphasizes the efficacy of ExCR on improving QoL and exercise capacity in patients with HF. Taylor et al., through an IPD meta-analysis of 3990 patients, predominantly with reduced ejection fraction HF, found significant benefits of ExCR, noting a 21.0 m improvement in the 6MWT and a 5.9-point enhancement in the Minnesota Living with HF score. Piotrowicz et al.’s comparison between home-based telemonitored cardiac rehabilitation and standard outpatient-based rehabilitation across 131 patients highlighted significant QoL improvements in both groups, with distinct benefits in mental health for home-based rehabilitation and physical well-being for standard rehabilitation. Our review corroborates these findings by demonstrating the variability in exercise training’s impact on perceived QoL, evidenced by significant improvements in KCCQ scores and 6MWT outcomes, and underscores the importance of personalized exercise regimens, particularly the potential superiority of HIIT in enhancing patient-perceived QoL. Even though hydration status changes were not described here, it is also important to recognize that their impact on cardiovascular risk factors in heart failure patients highlights the need for future research to include these parameters for a more holistic assessment.

Brubaker et al. reported significant enhancements in physical function measures, with VO2peak and 6MWT outcomes showing notable improvements in the endurance exercise training group compared to controls after 16 weeks of 3 times weekly exercises [33]. However, their study, focusing on HFpEF patients, did not find significant correlations between physical function improvements and changes in QoL measures, suggesting independent pathways of benefit. Slimani et al. [34], through a meta-analysis involving 2409 patients, quantified exercise training’s effects, showing a small effect size (ES) of −0.69 on total QoL and moderate ES of 0.91 on cardiac function, indicating substantial benefits across these parameters. Notably, resistance training emerged as the most effective mode, with an ES of 1.71 for enhancing aerobic capacity, followed by aerobic training (ES = 0.51). 

Heart failure is a chronic condition that is frequently associated with a multitude of comorbidities. Therefore, exploring the QoL in these patients requires the study and comparison of various domains and comorbid conditions. For example, patients with cardiac resynchronization can be considered as a separate study direction in QoL assessment. Guo et al. [35] found that in CHF patients undergoing cardiac resynchronization therapy, non-HIIT significantly improved maximal workload by 26.32 W, exercise duration by 68.95 s, peak oxygen uptake (VO2) by 3.05 mL/kg/min, left ventricular ejection fraction (LVEF) by 4.97%, and HRQoL, with a notable decrease in MLHFQ scores by 19.96. In contrast, Tegenge et al.’s broader analysis across various ExCR delivery modes showed VO2peak improvements following center-based (3.10 mL/kg/min), home-based (2.69 mL/kg/min), and technology-enabled ExCR (1.76 mL/kg/min), alongside reductions in HF-related hospitalization and mortality risks only after center-based ExCR (OR = 0.41 for hospitalization and OR = 0.42 for mortality) [36]. Similarly, Murray et al. [27] identified a significant improvement among patients with DM as well, a very common comorbidity correlated with HF and other pathologies [37,38]. 

The studies by Yao et al. [39] and Ostman et al. [40] provide insightful analyses into the effects of traditional Chinese exercise and varying exercise training intensities on patients with HF, respectively. Yao et al.’s meta-analysis of 721 patients revealed that TCE significantly enhances motor function and endurance (mean difference (MD) = 68.23), improves QoL (MD = −9.51), reduces plasma B-type natriuretic peptide levels (MD = −59.77), and decreases hospitalizations and associated costs (MD = −0.83 and MD = −1.6, respectively), suggesting traditional Chinese exercise as an effective adjuvant therapy for HF patients. However, no significant changes were noted in left ventricular ejection fraction and maximal oxygen consumption. Conversely, Ostman et al., through a review of 25 studies involving 2385 participants, demonstrated that higher exercise training intensities lead to greater improvements in QoL, with significant reductions in the MLWHF total score after high- (MD = −13.74) and vigorous-intensity training (MD = −8.56), but not moderate-intensity. Their findings suggest that aerobic or combined aerobic and resistance training may offer the most substantial benefits. Our systematic review aligns with these findings by highlighting the variability in exercise training’s impact on HF patients’ QoL and physical function. While Yao et al. [39] underscore the benefits of TCE in improving several outcomes without enhancing cardiac function directly, Ostman et al. reveal the potential for exercise intensity to dictate the magnitude of QoL improvements. The studies by Calabrese et al. [41] and Zhang et al. [42] delve into novel therapeutic strategies for patients with HF in the context of COVID-19, each exploring different rehabilitation modalities. Calabrese et al. [41] emphasize the cardiovascular complications associated with COVID-19, such as HF and thromboembolism, and advocate for exercise training as a critical component of cardiac rehabilitation. They highlight versatility and adaptability of exercise training to patient needs, capable of improving cardiovascular health and aiding in the recovery from endothelial dysfunction and thromboembolic complications. On the other hand, Zhang et al. [42] explore the safety and efficacy of digital-therapeutics-based cardiac rehabilitation, a novel approach necessitated by the difficulties in providing center-based cardiac rehabilitation during the pandemic. Their analysis, involving more than a thousand patients, demonstrates that digital therapeutics, including medical applications and wearable devices, can significantly enhance exercise capacity and QoL, with high adherence rates and no serious adverse events reported. Our systematic review aligns with these findings, suggesting that both traditional exercise training and digital therapeutics offer viable pathways for cardiac rehabilitation in patients with HF post-COVID-19. While Calabrese et al. provide evidence for the physical benefits of tailored ET programs, Zhang et al. [42] present a promising alternative through digital platforms, especially during times when conventional center-based rehabilitation is not feasible.

The potential applicability of both traditional exercise training and digital therapeutics in cardiac rehabilitation could indeed transcend the HF and post-COVID-19 conditions, extending to post-transplant patients and individuals with chronic metabolic diseases [43,44]. Such populations often face unique health challenges and complications that could benefit from the adaptable and patient-centered approaches highlighted by Calabrese et al. [41] and Zhang et al. [42].

It is also imperative to recognize that HF often coexists with other comorbid conditions, such as chronic obstructive pulmonary disease (COPD), which is among the most prevalent and significantly impacts patients’ QoL and rehabilitation outcomes [45]. The interplay between HF and COPD necessitates a comprehensive approach to cardiac rehabilitation that not only addresses the cardiovascular sequelae of diseases like COVID-19 but also considers the compounded effects of coexisting conditions [46]. As studies by Calabrese et al. and Zhang et al. [41,42] suggest, tailored exercise programs and innovative digital therapeutics offer promising strategies for improving cardiovascular health and overall QoL. However, the success of these interventions depends on a holistic treatment model that integrates management strategies for concurrent comorbidities, underscoring the importance of personalized care plans in optimizing rehabilitation and enhancing the QoL for patients with HF with complex health profiles. 

The KCCQ offers a unique advantage in its sensitivity to changes in the QoL specifically related to HF, which distinguishes it from other QoL measures. This specificity makes it particularly effective in documenting the impact of physical exercise on patients with HF. Regarding the relationship with counter-resistance exercise, research indicates that this form of training can significantly improve muscular strength and endurance, which are essential for daily activities and overall well-being in patients with HF [47]. The use of the KCCQ allows for a detailed assessment of these improvements, highlighting the tangible benefits of counter-resistance exercises in this patient population.

### 4.2. Limitations

The primary limitations stem from the heterogeneity of the included studies in terms of patient populations, intervention modalities, and outcome measures, particularly the KCCQ scores. The variability in HF etiology, severity, and the diverse exercise training programs (ranging from high-intensity interval training to telemedicine-based interventions) complicates direct comparison of their impact on QoL. Additionally, the reliance on self-reported QoL measures, while valuable, introduces subjectivity and potential bias in assessing the intervention’s efficacy. These factors collectively challenge the generalization of findings and underscore the need for more standardized and objective methodologies in future research to accurately determine exercise rehabilitation’s effects on the QoL of patients with HF.

## 5. Conclusions

The KCCQ’s effectiveness is highlighted by its ability to detect clinically meaningful improvements in QoL across diverse exercise modalities, including HIIT and MCT, tailored to the specific needs of heart failure populations. The consistent correlation between KCCQ score improvements and enhanced physical outcomes, such as the 6MWT, supports its reliability in capturing the nuanced benefits of exercise interventions on patient well-being. This correlation not only justifies the KCCQ’s utility in clinical and research settings but also emphasizes its role in guiding the optimization of personalized exercise programs for heart failure patients.

## Figures and Tables

**Table 1:** Study characteristics.

Study and Author	Country	Study Year	Study Design	Quality of Evidence
1 [21] Norman et al.	United States	2012	Prospective cohort	Medium
2 [22] Kitzman et al.	United States	2016	Randomized trial	High
3 [23] Parikh et al.	United States	2016	Randomized trial	High
4 [24] Mueller et al.	Germany	2021	Randomized trial	High
5 [25] Kitzman et al.	United States	2021	Randomized trial	High
6 [26] Güder et al.	Germany	2021	Prospective cohort	Medium
7 [27] Murray et al.	United States	2021	Randomized trial	High
8 [28] Chen et al.	Taiwan	2022	Retrospective cohort	Medium
9 [29] Nagatomi et al.	Japan	2022	Randomized trial	Medium

**Table 2:** Characteristics of the participants.

Study Number	Age (years)	Sex (Men)	Intervention Group	Control Group	LVEF	BMI (kg/m^2^)	Natriuretic Peptides
1 [21] Norman et al.	Intervention: 56.0 Control: 63.0	Intervention: 12 (60.0%) Control: 11 (55.0%)	20 with HF	20 with HF attention control	Intervention: 34.0% Control: 32.3%	Intervention: 33.0 Control: 33.2	BNP (at finish) Intervention: 1.68 Control: 2.09
2 [22] Kitzman et al.	Intervention: 66.9 Control: 66.0	Intervention: 10 (20%) Control: 9 (18%)	51 obese with HFPEF	49 obese with HFpEF no training	HFpEF Intervention: 60% Control: 60%	Intervention: 40.3 Control: 38.4	BNP (baseline) Intervention: 24.9 Control: 21.6
3 [23] Parikh et al.	Intervention: 60 Control: 59	Intervention: 297 (73%) Control: 1373 (71%)	406 HF with angina	1925 HF without angina	Intervention: 25% Control: 25%	Intervention: 30 Control: 30	NT-proBNP Intervention: 716 Control: 839
4 [24] Mueller et al.	HIIT: 70 MCT: 70 Control: 69	HIIT: 17 (29%) MCT: 23 (40%) Control: 19 (32%)	58 with HF HIIT 58 with HF MCT	60 with HF no training	HIIT: NR MCT: NR Control: NR	HIIT: 30.0 MCT: 31.1 Control: 29.0	NT-proBNP (baseline) HIIT: 475 MCT: 656 Control: 875
5 [25] Kitzman et al.	Intervention: 73.1 Control: 72.2	Intervention: 90 (51.4%) Control: 76 (43.6%)	175 with HF	174 with HF attention control	(≥45%) Intervention: 93 (53%) Control: 92 (53%)	Intervention: 32.9 Control: 33.0	BNP (baseline) Intervention: 595 Control: 645 NT-proBNP (baseline) Intervention: 2527 Control: 3615
6 [26] Güder et al.	64	9 (75%)	12 with HF	No	36%	29.8	NT-proBNP (baseline) 985
7 [27] Murray et al.	Intervention DM: 72.9 Control DM: 70.9 Intervention: 73.3 Control: 73.5	Intervention DM: 55 (53.3%) Control DM: 35 (42.1%) Intervention: 35 (66.9%) Control: 41 (55.1%)	103 with HF and DM 72 with HF	83 with DM attention control 91 without DM attention control	(≥45%) Intervention DM: 63 (61.2%) Control DM: 43 (51.8%) Intervention: 30 (41.7%) Control: 49 (53.8%)	Intervention DM: 34.3 Control DM: 34.7 Intervention: 30.8 Control: 31.4	BNP (baseline) Intervention DM: 383 Control DM: 473 Intervention: 759 Control: 673
8 [28] Chen et al.	Intervention: 57.7 Control: 58.8	Intervention: 321 (84.3%) Control: 111 (81.0%)	137 with HF <40% EF	381 with HF <40% EF no training	Intervention: 29.0 Control: 29.3	Intervention: 26.0 Control: 25.7	NR
9 [29] Nagatomi et al.	Intervention: 59.8 Control: 67.7	Intervention: 9 (60%) Control: 7 (47%)	15 with HF HBCR	15 with HF	Intervention: 39.9 Control: 44.5	Intervention: 20.2 Control: 21.1	BNP (baseline) Intervention: 237 Control: 192

NR—not reported; HF—heart failure; LVEF—left ventricle ejection fraction; BMI—body mass index; BNP—brain natriuretic peptide; HFpEF—heart failure with preserved ejection fraction; NT-proBNP—N-terminal prohormone of brain natriuretic peptide; HIIT—high-intensity interval training; MCT—moderate continuous training; DM—diabetes mellitus; HBCR—home-based cardiac rehabilitation.

**Table 3:** Rehabilitation program characteristics.

Study Number	Training Time, Follow-Up	Frequency of Training	Rehabilitation Program Description
1 [21] Norman et al.	24 weeks	Aerobic: 3 days per week; 30 min + 15-min warm-up + 15-min cool-down; Resistance training: 2 days per week	Aerobic: 40% to 70% HRR or RPE 11–14 on the Borg scale; Resistance training: 8 to 10 exercises (upper and lower extremity), 1 set of 10 to 15 repetitions.
2 [22] Kitzman et al.	20 weeks	Aerobic: 3 days per week	Primarily walking, with individualized prescriptions based on test results.
3 [23] Parikh et al.	2.5 years	≥90 min weekly exercise during months 1–3 and ≥120 min weekly thereafter	36 supervised sessions followed by 2 years of home-based training.
4 [24] Mueller et al.	12 months	HIIT: 3 days per week MCT: 5 days per week	HIIT: 10-min warm-up at 35–50% HRR, followed by 4 × 4-min intervals at 80–90% HRR, with 3 min of active recovery between intervals. MCT: 40 min at 35–50% HRR.
5 [25] Kitzman et al.	3 months	Aerobic: 3 days per week	Facility-based: 36 sessions of 60 min over 12 weeks. Home-based: 30 min daily (low-intensity walking and strengthening exercises).
6 [26] Güder et al.	12 months	Group session: once per week Aerobic: daily	Group sessions: training intensity adjusted to target heart rate from CPET at 70% of peak VO2 ± 10 beats per minute; training goal was moderate intensity targeted (Borg’s scale 11–13). Session duration: 60 min; Aerobic: at least 60 min per day.
7 [27] Murray et al.	3 months	Aerobic: 3 days per week	Aerobic: 60 min three times weekly, focusing on strength, balance, mobility, and endurance. Home exercise: low-intensity walking and strengthening exercises on non-intervention days, after a safety check by study staff.
8 [28] Chen et al.	3 months	3 days per week	Aerobic: 40 min sessions treadmill walking/jogging, cycling, stair climbing, elliptical training. Target intensity: 40–60% of peak oxygen consumption (VO2/kg) or 10 beats below CPET heart rate endpoints. Intensity increased fortnightly targeting Borg RPE of 12–14. Resistance exercise: 10–15 repetitions/set, 1–3 sets/session, 2–3 days/week after 4 weeks of moderate training. RPE intensity of 11–13. Flexibility exercises: As per ACSM guidelines, static stretches held for 10–30 s, advised to hold for at least 15 s with more than four repetitions.
9 [29] Nagatomi et al.	3 months	Aerobic: 3–5 times per week Resistance training: 2–3 times per week	Types of exercises: Stretching, resistance training (using weights), and aerobic exercises (ergometry or walking). Exercise intensity: Set at 11–13 on the Borg scale.

NR—not reported; HRR—heart rate reserve; RPE—rating of perceived exertion; HIIT—high-intensity interval training; MCT—moderate continuous training.

**Table 4:** KCCQ in association with 6MWT study outcomes.

Study Number	KCCQ (Baseline)	KCCQ (Finish)	6MWT (Baseline)	6MWT (Finish)	Significance
1 [21] Norman et al.	Intervention: 69.7 Control: 72.8	Intervention: 81.0 Control: 77.9	Intervention: 408 Control: 352	Intervention: 463 Control: 384	KCCQ—not statistically different between groups 6MWT—statistically different but no clinical significance
2 [22] Kitzman et al.	Intervention: 75 Control: 73	Difference (95% CI): 2 (−3, 7)	Intervention: 1503 Control: 1397	Difference (95% CI): 106 (60, 152)	KCCQ—not statistically different between groups 6MWT—statistically different
3 [23] Parikh et al.	Intervention: 60 Control: 70	No significant change	Intervention: 373 Control: 370	No significant change	KCCQ—not statistically different between groups 6MWT—not statistically different
4 [24] Mueller et al.	HIIT: 68.0 MCT: 62.2 Control: 65.7	HIIT: 80 MCT: 77 Control: 72	NR	NR	KCCQ—significantly higher in HIIT vs. MCT and controls
5 [25] Kitzman et al.	Intervention: 40 Control: 42	Intervention: 69 Control: 62	Intervention: 194 Control: 293	Intervention: 193 Control: 260	KCCQ—significantly higher and improved compared to controls 6MWT—significantly higher and improved compared to controls
6 [26] Güder et al.	62	73	450 m	470	KCCQ—not significantly improved 6MWT—not significantly improved
7 [27] Murray et al.	Intervention DM: 40.1 Control DM: 41.0 Intervention: 40.3 Control: 42.0	Intervention DM: 63.8 Control DM: 60.0 Intervention: 74.3 Control: 62.0	Intervention DM: 183 Control DM: 178 Intervention: 209 Control: 206	Intervention DM: 281 Control DM: 252 Intervention: 286 Control: 248	KCCQ—not significantly improved regardless of DM status 6MWT—significantly improved in both study groups
8 [28] Chen et al.	NR	Intervention: 32.9 points improvement Control: 20.3 points improvement	NR	NR	KCCQ—significantly improved
9 [29] Nagatomi et al.	Intervention: 70 Control: 74	Intervention: 70 Control: 78	NR	Intervention: +52.1 difference Control: −4.3 difference	KCCQ—not significantly improved 6MWT—significantly improved

KCCQ—Kansas City Cardiomyopathy Questionnaire; 6MWT—6 Minute Walk Test; CI—confidence interval; NR—not reported; HIIT—high-intensity interval training; MCT—moderate continuous training.

## Data Availability

Not applicable.
